# Molecular Bases Underlying the Hepatoprotective Effects of Coffee

**DOI:** 10.3390/nu9010085

**Published:** 2017-01-23

**Authors:** Federico Salomone, Fabio Galvano, Giovanni Li Volti

**Affiliations:** 1 Division of Gastroenterology, Ospedale di Acireale, Azienda Sanitaria Provinciale di Catania, 95124 Catania, Italy; 2 Department of Biomedical and Biotechnological Sciences, University of Catania, 95125 Catania, Italy; fgalvano@unict.it (F.G.); livolti@unict.it (G.L.V.)

**Keywords:** caffeine, chlorogenic acid, liver fibrosis, liver steatosis, liver cancer

## Abstract

Coffee is the most consumed beverage worldwide. Epidemiological studies with prospective cohorts showed that coffee intake is associated with reduced cardiovascular and all-cause mortality independently of caffeine content. Cohort and case-control studies reported an inverse association between coffee consumption and the degree of liver fibrosis as well as the development of liver cancer. Furthermore, the beneficial effects of coffee have been recently confirmed by large meta-analyses. In the last two decades, various in vitro and in vivo studies evaluated the molecular determinants for the hepatoprotective effects of coffee. In the present article, we aimed to critically review experimental evidence regarding the active components and the molecular bases underlying the beneficial role of coffee against chronic liver diseases. Almost all studies highlighted the beneficial effects of this beverage against liver fibrosis with the most solid results indicating a pivot role for both caffeine and chlorogenic acids. In particular, in experimental models of fibrosis, caffeine was shown to inhibit hepatic stellate cell activation by blocking adenosine receptors, and emerging evidence indicated that caffeine may also favorably impact angiogenesis and hepatic hemodynamics. On the other side, chlorogenic acids, potent phenolic antioxidants, suppress liver fibrogenesis and carcinogenesis by reducing oxidative stress and counteract steatogenesis through the modulation of glucose and lipid homeostasis in the liver. Overall, these molecular insights may have translational significance and suggest that coffee components need clinical evaluation.

## 1. Introduction

Coffee is the most consumed beverage worldwide with an impressive impact on the economy of producing countries. Coffee is prepared from the seeds of the coffee plant, genus *Coffea*, a member of the Rubiaceae family, which includes hundreds of different species. Commercial production mainly exploits the seeds of *Coffea arabica* (arabica coffees), which represent about 70% of the world market, while *Coffea canephora* (commonly known as robusta coffees), which has a more bitter taste than arabica, is used principally for instant and espresso coffees (www.ico.org).

**Figure 1:**
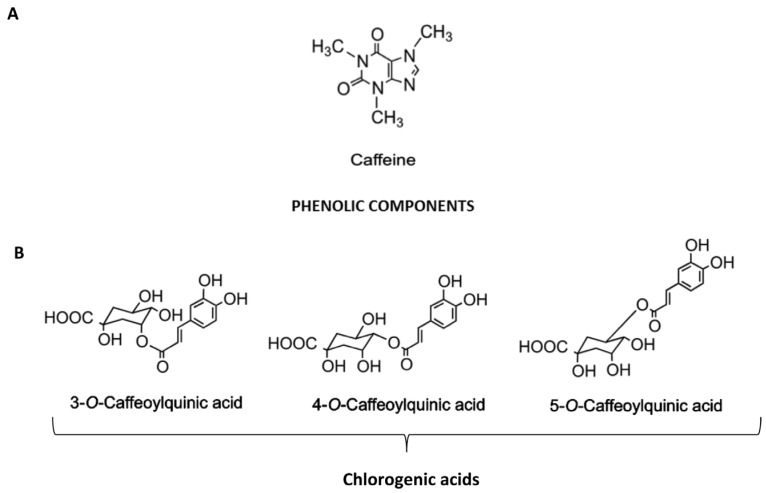
Chemical structure and cellular targets of coffee hepatoprotective components. (**A**) Caffeine is an alkaloid belonging to the methylxanthines family; (**B**) Chlorogenic acids belong to conjugated hydroxycinnamates, a family of non-flavonoid phenols formed by a single phenolic ring linked to three carbons. The main CGAs are 5-*O*-caffeoylquinic acid (5-CQA) and its isomers 3-*O*-caffeoylquinic acid (3-CQA) and 4-*O*-caffeoylquinic acid (4-CQA).

The association between coffee consumption and all-cause and cause-specific mortality, especially cardiovascular mortality, has been investigated in independent studies evaluating prospective cohorts, with most solid studies finding an inverse association with all-cause and cardiovascular mortality for both caffeinated and decaffeinated blends [1,2]. Recently, the inverse association between coffee consumption and all-cause, cardiovascular and cancer mortality was confirmed by a meta-analysis [3]. The impact of coffee intake on chronic liver diseases has been a matter of debate in the last two decades, with some conflicting results due the retrospective nature of most of the studies [4]. Recently, a well-designed, population-based, prospective cohort study of >215,000 men and women, with a 18-year follow-up period, showed reduced risk of incident hepatocellular carcinoma (HCC) and chronic liver disease mortality in those who consume high levels of coffee, regardless of the participants’ ethnicity, sex, body mass index, smoking status, alcohol intake, or diabetes status [5]. Throughout the last years, in parallel with epidemiological data, several experimental in vitro and in vivo studies have provided biological plausibility for the hepatoprotective effects of whole coffee or of specific compounds, with most studies evaluating the effects of caffeine (Figure 1A) and of chlorogenic acids (CGA), a family of conjugated hydroxycinnamates (Figure 1B).

In this review, we aimed to summarize experimental evidence regarding molecular mechanisms underlying the hepatoprotective effects of coffee by reviewing in vitro and in vivo studies. A search of the electronic databases Medline and Embase was performed to retrieve relevant articles published up to May 2016. A combination of the keywords “coffee”, “caffeine”, “coffee polyphenols“, “chlorogenic acid”, “cafestol”, “kahweol”, “melanoidins”, “liver steatosis”, “liver damage”, “liver injury”, “liver inflammation”, “liver fibrosis”, “liver cirrhosis”, “liver cancer” was searched. All published studies in the English language were considered. Additional papers were retrieved by references of epidemiological studies on coffee and liver diseases. After careful evaluation, the following studies were excluded from the review: (i) studies reporting in vivo effects on acute models of liver damage or failure (acetaminophen, etc.); (ii) studies reporting in vitro effects on metabolic processes without assessing effects on cell steatosis or apoptosis (pure metabolic studies).

## 2. Coffee and Liver Steatosis

Convincing results from a meta-analysis of 28 prospective studies have indicated that both caffeinated and decaffeinated coffee consumption is associated with lower risk of diabetes in a dose-response manner [6]. Consistently, a recent systematic review of seven studies assessing the association of non-alcoholic fatty liver disease (NAFLD) with coffee consumption found that higher coffee intake is associated with a decreased risk of fibrosis related to NAFLD but not with a lower prevalence of liver steatosis [7], although only one study had a prospective design [8]. At the molecular level, the effects of coffee on liver steatogenesis have been extensively studied in in vitro and in vivo models (Table 1).

To our best knowledge, the first experimental study showing a protective effect of a coffee component against liver steatosis was from Rodriguez de Sotillo and Hadley [16], who showed that genetically obese Zucker rats treated with 5 mg/kg bw of CGA for three weeks via intravenous infusion displayed improved glucose tolerance and reduced plasma and liver triglycerides; however, in this study, the results were obtained in a genetic model of obesity and no molecular pathway related to CGA effects was identified. Successively, Shimoda et al. [17] evaluated the effects of the oral administration of green coffee bean extract vs. caffeine vs. CGA in mice fed a standard diet for two weeks and showed decreased levels of hepatic triglycerides in the CGA groups, although experimental findings were obtained not in a model of obesity. The effects of CGA administration on glucose production and fatty acid synthesis have been also assessed in genetically obese db/db mice by Ong et al. [12], demonstrating that chronic administration of CGA attenuates hepatic steatosis and improves glucose tolerance and insulin sensitivity; interestingly, in this study, inhibition and knockdown of AMP-activated protein kinase (AMPK) abrogated the beneficial effects of CGA, thus indicating that CGA improved glucose and lipid metabolism, via the activation of AMPK. Consistent with these results, in an elegant study, Murase et al. [14] assessed the effects of the coffee polyphenolic fraction, which includes CGA and similar compounds, in mice fed a High Fat Diet (HFD) for 15 weeks; the authors showed that the adjunct of coffee polyphenols to HFD completely reversed the metabolic disturbances associated with HFD-induced obesity. In particular, feeding coffee polyphenols (CPP) counteracted body weight gain, visceral fat accumulation and liver steatogenesis by enhancing the whole-body metabolic rate, without affecting food intake, locomotor activity or fecal lipid excretion; CPP decreases the expression of lipogenic enzymes such as fatty acid synthase (FAS), acetyl-CoA carboxylase-1 (ACC1) and stearoyl-CoA desaturase-1 (SCD1) and its transcription factor, sterol regulatory element-binding protein 1-c (SREBP1-c), in the liver and adipose tissue. CPP suppressed the expression of lipogenic enzymes also in Hepa 1–6 cells, thus indicating that CPP are potent modulators of lipid homeostasis in the hepatocytes. These studies pointed out the effects of coffee polyphenols on steatosis and metabolism; however, there is also evidence on the effect of coffee on inflammation and fibrosis related to NAFLD. Similarly, Vitaglione et al. [15] reported that consumption of decaffeinated espresso coffee is able to reduce not only liver steatosis but also inflammation and fibrosis in rats fed a HFD for 12 weeks, underlining for the first time that caffeine is not essential for the anti-fibrotic effects of coffee and that specific coffee components, i.e., coffee polyphenols and melanoidins, contribute to the hepatoprotective effect. In particular, the intake of coffee polyphenols recapitulates the effects of whole coffee in counteracting oxidative stress, inflammatory cytokine imbalance and fibrogenesis [15]. Successively, Ma et al. [9] assessed both the preventive and therapeutic effects of CGA in mice fed a HFD and showed that the preventive administration of CGA blocked the development of fatty liver without affecting body weight and CGA treatment reversed HFD-induced hepatic steatosis and insulin resistance by suppressing the hepatic expression of the peroxisome-proliferator activated receptor gamma (*Pparγ*), cluster of differentiation (*Cd36*), fatty acid binding protein 4 (*Fabp4*) and *Mgat1* genes; more interestingly, CGA treatment also attenuated inflammation in the liver and white adipose tissue accompanied by a decrease in the mRNA levels of macrophage marker genes including EGF-like module-containing mucin-like hormone receptor-like 1 (*F4/80*), cluster of differentiation (*Cd68*), integrin *Cd11b*, integrin *Cd11c* and tumor necrosis factor alpha (*Tnfα*), monocyte chemotactic protein 1 (*Mcp-1*) and C-c chemokine receptor type 2(*Ccr2*) encoding inflammatory proteins.

Due to the tight connection of NAFLD with cardiovascular mortality, it is important to highlight the results from Panchal et al. [13], who evaluated the effects on cardiovascular and liver damage of an eight-week treatment with Colombian coffee extract in rats fed a high-sucrose, high-fructose and high-fat diet for 16 weeks; in this study, the administration of coffee extract reduced steatosis, inflammation and portal fibrosis and counteracted the development of cardiovascular remodeling without reducing body weight gain [13]. Strikingly, in this study, the dose of the coffee extract administered to rats was of 3 g/kg of body weight which corresponds to 35 g/day in a 70 kg man, based on body surface area comparisons between rats and humans [18], i.e., four to five cups per day, which is similar to doses that decreased the risk of significant fibrosis in human NAFLD [7].

Recently, further studies suggested the impact of coffee components not only on metabolic pathways in the liver but also on endoplasmic reticulum stress and autophagy, which are pivot processes in NAFLD [19]. Our group assessed the effects of decaffeinated coffee on rats fed HFD and showed that coffee intake reduces liver injury not only by decreasing oxidative stress, as shown by reduced isoprostanes and 8-deoxyguanosine, but also by modulating the expression of cell chaperones such as glucose-regulated protein (GRP-78), which is involved in correct protein folding, and protein deglycase (DJ-1), which regulates autophagy [11]. In an elegant study, using genetic, pharmacological, and metabolomic approaches, Sinha et al. [10] showed that caffeine is a potent stimulator of hepatic autophagic flux; caffeine-induced autophagy involves the down-regulation of mammalian target of rapamycin signaling and alterations in hepatic amino acids and sphingolipid levels. In mice fed a HFD, caffeine markedly reduced hepatosteatosis and concomitantly increased autophagy and lipid uptake in lysosomes.

## 3. Coffee and Liver Fibrosis/Cirrhosis

The association of coffee consumption with fibrosis and cirrhosis development has been investigated in several studies [4]. In a prospective study of patients with hepatitis C and bridging fibrosis or cirrhosis, baseline coffee intake was inversely associated with liver disease progression [20]. In a randomized controlled trial, the administration of four cups of coffee for 30 days reduced 8-hydroxydeoxyguanosine and collagen levels [21]. However, the anti-fibrotic effect of coffee is independent from the etiology of liver disease. In fact, a recent meta-analysis by Liu quantitatively assessed results from 16 studies involving 3034 coffee consumers and 132,076 non-consumers; pooled results of the meta-analysis indicated a protective effect of coffee against cirrhosis and advanced fibrosis of any etiology. In particular, the odds ratio (OR) of developing cirrhosis was 0.61 in patients with a coffee intake ≥2 cups versus non-consumers, whereas the OR of developing advanced fibrosis was 0.73 in patients with a coffee intake ≥2 cups versus non-consumers. In agreement with epidemiological results, several experimental studies have suggested that specific coffee components, i.e., caffeine and the polyphenolic fraction, are able to modulate molecular pathways related to fibrogenesis (Table 1).

Coffee has been consumed for several centuries worldwide for its flavor and taste but also because of the psychoactive effects of caffeine, an alkaloid which is contained in different quantities according to coffee species, techniques of roasting and brewing [22,23]. To the best of our knowledge, the first experimental study that showed a protective effect of a coffee component against liver fibrosis was from Chan et al., who showed that caffeine reduces fibrosis in mice chronically treated with carbon tetrachloride (CCl_4)_ or thioacetamide and attributed this effect to its nonselective adenosine receptor antagonist activity [24] (Table 2). Successively, Gressner et al. [25] showed that caffeine inhibits TGF-beta–induced connective tissue growth factor (CTGF) expression in hepatocytes by the stimulation of the degradation of the TGF-beta effector mothers against homolog decapentaplegic 2 (SMAD2), the inhibition of mothers against homolog decapentaplegic 3 (SMAD3) phosphorylation and the up-regulation of the (PPARgamma) receptoy. The same authors demonstrated that the caffeine metabolite paraxanthine is able to down-regulate the expression of the fibrogenic protein CTGF in hepatic stellate cells [26] and to reduce liver fibrosis and lipid peroxidation in rats with bile duct ligation (BDL) [27].

Arauz et al. and Furtado et al. [35,36] independently reported the effects of whole coffee vs. decaffeinated coffee in rats treated with thioacetamide, showing that both types of coffee reduce liver fibrosis and TGF-β expression. Interestingly, Gordillo-Bastida et al. [31] not only confirmed the inhibitory effects on thiocatemide-induced fibrosis but revealed that caffeine administration is associated with increased activity of superoxide dismutase and catalase in the liver and increased expression of nuclear factor E2-related factor 2 (Nrf2), the prototypical transcription factor inducing antioxidant enzymes.

The anti-fibrotic effects of caffeine have been also evaluated in in vitro cultures of human and rodent hepatic stellate cells. Shim et al. assessed the effect of caffeine on human hepatic stellate cells (HSC) proliferation and migration and found that caffeine attenuates the progression of liver fibrosis by inhibiting HSC adhesion and activation [32]. Wang et al. assessed the effects of caffeine in an immortalized rat HSC line treated with acetaldehyde, an in vitro model of alcohol-induced fibrosis, and demonstrated that caffeine suppresses the production of procollagen I and of procollagen III via inhibition of the cAMP-PKA-SRC-ERK1/2 and P38 MAPK pathways, respectively [30]. These effects were also reproduced in primary rat HSCs and in an in vivo model of alcoholic fibrosis by the same group [29].

Beside morphological evidence for the anti-fibrotic activity of caffeine coming from the mentioned studies, recently it was elegantly shown by Hsu et al. that caffeine may also favorably impact hemodynamic changes in a rat model of portal hypertension [28]. In this study, Sprague-Dawley rats with common bile duct ligation–induced cirrhosis received a prophylactic or therapeutic caffeine treatment for four weeks. Compared to vehicle, caffeine 50 mg/kg/day both prophylactically and therapeutically decreased the cardiac index, increased systemic vascular resistance, and reduced portal pressure, portosystemic shunting and intrahepatic angiogenesis [28]. Consistently, prophylactic and therapeutic caffeine treatment decreased portal resistance and portal pressure in thioacetamide-induced cirrhotic rats. Caffeine down-regulated endothelial nitric oxide synthase, vascular endothelial growth factor (VEGF), phospho-VEGFR2, and phospho-Akt mesenteric protein expression. Caffeine adversely affected the viability of hepatic stellate and sinusoidal endothelial cells but did not modify the vascular response to vasoconstrictors in splanchnic, hepatic, and collateral vascular beds. The beneficial effects of caffeine were reversed by selective adenosine A1 agonist or A2A agonists, thus confirming that the mechanism of the hepatoprotection of caffeine is dependent on blocking adenosine receptors.

Although the anti-fibrotic effects of caffeine are well characterized, few experimental studies have been conducted so far to investigate the effects of polyphenols, such as CGA, in in vitro and in vivo models of liver fibrosis. Shi et al. first explored the effect of the oral administration of CGA in rats with CCl_4_-induced cirrhosis; the authors found that CGA reduces liver fibrosis and the expression of collagen I and collagen III. Consistently, rats treated with CGA displayed reduced levels of VEGF, TGF-beta and alpha-smooth muscle actin, thus indicating that CGA is able to counteract liver fibrogenesis in rats [37]. Successively, the same authors further extended their findings on the anti-fibrotic effects of CGA in the same experimental model by showing that CGA treatment reduces the expression of inflammatory cytokines, toll-like receptor 4 (TLR4), myeloid differentiation factor 88, inducible nitric oxide synthase and cyclooxygenase-2 and nuclear factor-κB activation [33]. These findings were replicated on lipopolysaccharide (LPS)-induced activation in rat hepatic stellate cells, in which the treatment of CGA can inhibit LPS-induced production of reactive oxygens species (ROS), nuclear translocation of NF-κB and IκB-α phosphorylation in HSCs and can down-regulate the secretion of MCP-1 and IL-6 in the culture supernatant [34].

## 4. Coffee and Hepatocellular Carcinoma

The epidemiological association between coffee intake and liver cancer prevalence or incidence has been extensively studied throughout the two last decades [4] (Table 3). A meta-analysis of cohort and case-control studies, updated up to September 2012, showed that the risk of HCC is reduced by 40% for any coffee consumption vs. no consumption [38]. Recently, convincing results on the protective effects of coffee against liver cancer development were obtained in a well-designed prospective study [5].

At the experimental level, different studies have been conducted so far to identify the molecular determinants of such effects. To our best knowledge, the first experiment that showed the beneficial effects of a coffee component on liver carcinogenesis was conducted by Mori H et al. [45], who reported that a diet containing 0.025% CGA for 24 weeks in Syrian golden hamsters is able to reduce the numbers of hyperplastic liver cell foci in a chemical model of colon and liver cancer induced by the intravenous injection of methylazoxymethanol acetate. The same research group further extended these results by showing decreased incidences of liver tumors in rats given a concurrent administration of aminopyrine (0.01%) and sodium nitrite (0.1%) and coffee solution as drinking water for 630 days [41]. Successively, Miura Y et al. showed that sera from rats administered instant coffee powder (ICP) suppress in vitro [40] the proliferation and invasion of AH109A, a rat ascites hepatoma cell line, whereas dietary ICP consistently significantly reduces solid tumor growth and tends to reduce hepatoma metastases to the lung and lymphatic nodes in vivo in hepatoma-bearing rats fed a 20% casein diet alone or supplemented with 0.1% ICP for two weeks [39].

Katayama et al. assessed the effects of coffee in a liver cancer–prone Long Evans Cinnamon rat and showed that coffee administration for 25 weeks delayed the occurrence of hepatitis, significantly improved survival, reduced the expression of inflammatory cytokines, and reduced the incidence of small pre-neoplastic liver foci in Long-Evans Cinnamon (LEC) rats [44]. Similar results were reported in two further models of liver cancer in rodents. Ferk et al. [43] showed reduced numbers and areas of hepatic preneoplastic foci in rats that consumed different brews of coffee and were subsequently treated with aflatoxin B1. Furtado et al. evaluated the effects of coffee or caffeine in a rat model of fibrosis/carcinogenesis induced by diethylnitrosamine (DEN) and CCl_4_. One week after DEN injection, the groups started receiving coffee or 0.1% caffeine ad libitum for 24 weeks. The groups receiving coffee or caffeine alone not only had a reduction in collagen content but also displayed a significant reduction in the size and area of pre-neoplastic lesions and in the mean number of neoplastic lesions [42].

As concerns the molecular targets involved in the chemopreventive effects of coffee, evidence points out the importance of Nrf-2. Cavin et al. [46] showed a coffee-dependent induction of enzymes involved in xenobiotic detoxification processes and in cellular antioxidant defenses; using an Antioxidant Responsive Element (ARE) ARE-reporter luciferase assay, they showed that these inductions are correlated with the activation of the Nrf2 transcription factor. Higgins et al. [47] extended these results by showing that a diet containing 6% coffee increases gene expression of hepatic and intestinal NAD(P)H:quinone oxidoreductase 1 (NQO1), glutathione S-transferase class Alpha 1 (GSTA1), intestinal Uridine-5′-diphosphoglucose (UDP)-glucuronosyl transferase 1A6 (UGT1A6) and the glutamate cysteine ligase catalytic (GCLC) subunit, and these inductions were partly abolished in mice lacking Nrf2. In an elegant study, Kalthoff S et al. [48] showed that the incubation of hepG2 and CaCo2 with coffee induces the transcription of several UDP glucuronosyltransferases (UGT1A), which are proteins with potent genoprotective capabilities, independently of caffeine, methylxanthines, or cafestol and kahweol content; the induction of UGT1A by coffee is regulated by the aryl hydrocarbon receptor (AhR) and Nrf2 by cis-acting antioxidant and xenobiotic response elements (ARE/XRE). An another study in rats fed a standard diet and drinking 2 mL/day of coffee for four weeks, Vicente et al. [49] showed increased activities of hepatic superoxide dismutase, catalase, and glutathione peroxidase and enhanced expression of cytosolic Nrf2, further confirming that the antioxidant effects of coffee in the liver are associated with Nrf2 signaling.

## 5. Nutraceutical/Pharmaceutical Perspective

**Figure 2:**
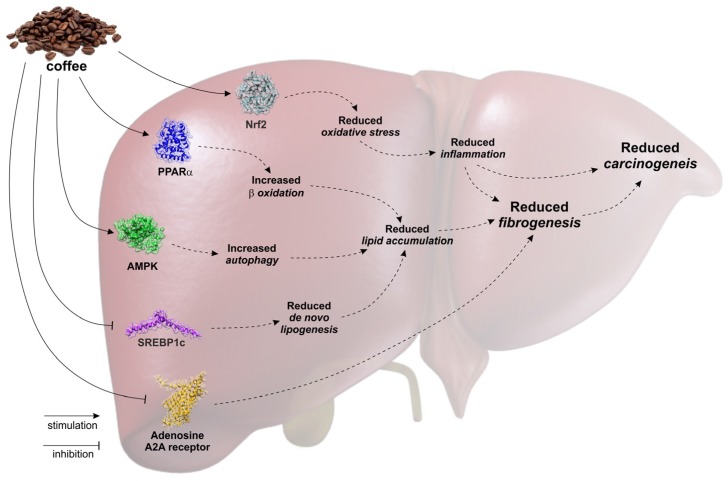
The bioactive components of coffee, caffeine and chlorogenic acids, inhibit de novo lipogenesis, promotes lipid oxidation and induces autophagy, thus reducing hepatocyte steatosis. Furthermore, chlorogenic acids reduce oxidative stress by activating the Nrf2 response whereas caffeine blocks the adenosine receptor A2. Overall, modulation of these pathways suppresses the production of inflammatory cytokines and the activation of hepatic stellate cells leading to reduced fibrogenesis and carcinogenesis.

Several epidemiological studies have associated the consumption of coffee with a decreased risk of developing chronic liver diseases. Consistently, experimental data suggest that specific coffee components, in particular caffeine and CCA, favorably impact liver steatogenesis, fibrogenesis and carcinogenesis by acting on different molecular and cellular targets (Figure 2). Therefore, it is reasonable to hypothesize a nutraceutical or pharmaceutical use for these compounds. In this respect, although experimental studies provide biological plausibility for the use of coffee extracts or components in clinical settings, the administration of these compounds is not currently recommendable due to a lack of well-designed clinical trials (clinicaltrials.gov). Consequently, there is a need for well-designed randomized controlled trials (RCTs) further investigating the effect of different doses of coffee or coffee bioactive components on healthy individuals as well as on patient populations. A small RCT performed so far showed that coffee consumption attenuates hepatic insulin resistance but not the increase of liver triglycerides induced by fructose overfeeding in healthy men, although these results were obtained after only two weeks of treatment [50]. A further issue is the bioavailability of coffee polyphenols, which can be influenced by a variety of factors. Nardini et al. first observed an increase of conjugated caffeine acid in plasma after the ingestion of coffee [51]. Rechner et al. detected ferulic acid, isoferulic acid, dihydroferulic acid, 3-methoxy-4-hydroxybenzoic acid, hippuric acid and 3-hydroxyhippuric acid in urine from human subjects after three ingestions of two cups of coffee at 4 h intervals [52]. Monteiro et al. demonstrated the presence of unmetabolized CGA in human plasma after acute ingestion of coffee containing about 4 umol of CGA [53]. A recent study by Stalmach et al. indicated that about 30% of the CGAs are absorbed in the small intestine of ileostomy subjects and that in subjects with a functioning colon, about 70% of intake passes from the small to the large intestine [54]. Thus, the “pharmaceutical” design of CGA in order to allow adequate bioavailability remains a priority, whereas a main problem for the nutraceutical or pharmaceutical use of caffeine remains the potent psychostimulant effect which is not desirable in some patients. In conclusion, coffee is a complex beverage with potential health benefits, containing a variety of bioactive compounds such as caffeine and CGAs. Experimental findings may have translational significance and indicate the importance of developing pharmaceutically designed nutraceuticals or drugs containing coffee compounds for therapeutic perspectives.

## Figures and Tables

**Table 1:** Effects of coffee or coffee components in experimental models of liver steatosis.

Author, Year (Reference)	Coffee Compound and Schedule	Experimental Model	Main Findings
Ma Y, 2015 [9]	CGA, 100 mg/kg i.p. twice/wk	C57BL/six mice fed a HFD for 15 weeks and treated with CGA for all 15 weeks or last six weeks	↓ steatosis and insulin resistance in both preventive and therapeutic arms ↓ Pparγ, Cd36, Fabp4, and Mgat1 ↓ liver and visceral adipose tissue inflammation ↓ F4/80, Cd68, Cd11b, Cd11c, Tnfα, Mcp-1 and Ccr2
Sihna RA, 2014 [10]	Caffeine	C57BL/six mice fed a HFD for four weeks Hepg2 treated with oleic/palmitic acid and caffeine	↑ autophagic flux in the liver ↑ autophagy in hepatic cells ↓ hepatic steatosis
Salomone F, 2014 [11]	Espresso decaffeinated coffee	Wistar rats fed a HFD for 12 weeks and treated with 1.5 mL of decaffeinated espresso coffee (equivalent to six cups) for the last eight weeks	↑ GSH, PPAR-α ↓ 8-OGH ↓ α-SMA, TGF-β ↑ GSH, PPAR-α
Ong Kw, 2013 [12]	Chlorogenic acid	Db/db mice, Hepg2	↓ fasting glucose ↓ serum TC, TG ↓ serum FFA ↑ serum adiponectin ↓ liver TC, TG ↓ lipid accumulation in hepatic cells
Panchal SK, 2012 [13]	Colombian coffee extract	Wistar rats fed a Western diet for 16 weeks and treated with CE 50 mL/kg of chow (50 mL = 50 g of coffee/100 mL of hot water) for the last eight weeks	↓ steatosis, inflammation and fibrosis ↑ glucose tolerance ↓ cardiac fibrosis, hypertension
Murase T, 2011 [14]	Coffee polyphenols	C57BL/6J mice fed a HFD + CPP (0.5%, 1% of chow) for two, 15 weeks Hepa 1–6 cells treated with CPP for 24 h	↓ body and liver weight in mice fed 1% CGA group ↓ liver triglycerides and cholesterol in mice fed 1% CGA group ↓ SREBP-1c, FAS, ACC-1, ACC-2, SCD-1 in mice fed 1% CGA and Hepa1-6 cells
Vitaglione P, 2010 [15]	Espresso decaffeinated coffee, coffee polyphenols or coffee melanoidins	Wistar rats fed a HFD for 12 weeks and treated with 1.5 mL of decaffeinated espresso coffee (equivalent to six cups) for the last eight weeks	↓ steatosis, inflammation and fibrosis ↑ liver adiponectin receptor ↓ inflammatory cytokines and oxidative stress
Rodriguez de Sotillo DV, 2002 [16]	CGA	Zucker rats fed a standard diet, daily treated with 5 mg/kg bw of CGA for three weeks via intravenous infusion	↑ glucose tolerance ↓ plasma cholesterol and triglycerides ↓ liver triglycerides

**Table 2:** Effects of coffee or coffee components in experimental models of liver fibrosis/cirrhosis.

Author, Year	Coffee Component	Study Design	Main Findings
Hsu SJ, 2015 [28]	Caffeine 50 mg/kg daily	Sprague-Dawley rats with BDL for four weeks or treated with thioacetamide for eight weeks were administered caffeine at d1 or 15 of study period	↓ cardiac index, portal pressure and portosystemic shunting ↓ endothelial nitric oxide synthase, vascular endothelial growth factor (VEGF), phospho-VEGFR2
Wang Q, 2015 [29]	Caffeine 5, 10, 20 mg/kg daily	Sprague-Dawley rats treated with alcohol + 20 mg/kg caffeine Rat primary HSC treated with	↓ AST, ALT ↓ hyaluronic acid, laminin, N-terminal peptide of type III procollagen and type IV collagen ↓ cAMP-PKA-CREB
Wang H, 2014 [30]	Caffeine 0.5–8 mM	Rat HSC-T6 treated with 200 µM acetaldehyde for 24–72 h	↓ Cell viability ↓ procollagen I and III ↓ cAMP-PKA-SRC-ERK1/2 ↓ P38 MAPK
Gordillo-Bastidas D, 2013 [31]	Caffeine 15 mg/kg daily	Wistar rats treated with thioacetamide for seven weeks or BDL for 4 weeks	↓ CTGF, TGF-β, Col-1 ↓ IL-6, IL-1, TNF-α ↑ Nrf-2, SOD, CAT ↓ Snail-1
Shim SG, 2013 [32]	Caffeine 1, 5, 10 mmol	-HSC treated with caffeine -Sprague-Dawley rats treated with thioacetamide for eight weeks	↑ HSC apoptosis ↓ HSC procollagen type 1c, α-SMA ↓ liver TGF-β, α-SMA
Shi H, 2013 [33]	Chlorogenic acid 12.5, 25 and 50 lg/mL	Rat HSC activated by LPS 100 ng/mL treated with CA for 24 h	↓ ROS ↓IκB-α phosphorylation ↓ MCP-1, IL-6
Shi H, 2013 [34]	Chlorogenic acid	Sprague-Dawley, CCl4 + CGA for eight weeks	↓ AST, ALT ↓ collagen deposition ↓ α-SMA, collagen I ↓ TLR4
Furtado KS, 2012 [35]	-Conventional coffee -Decaffeinated coffee -Caffeine 0.1%	Wistar rats treated with thioacetamide for eight weeks	↓ AST, ALT ↓ collagen volume fraction ↓ TGF-β ↓ GST-P positive preneoplastic lesions (only in conventional coffee group)
Arauz J, 2013 [36]	-Conventional -Decaffeinated coffee	Wistar rats treated with thioacetamide for eight weeks	↓ AST, ALT ↓ collagen volume fraction ↓ TGF-β, CTGF, α-SMA, MMP-2
Klemmer I, 2011 [27]	Caffeine metabolite (paraxanthine) 1 mM	Sprague-Dawley rats treated with BDL	↓ Picrosirius red staining ↓ CTGF, SMAD2 ↓ malondialdehyde
Shi H, 2009 [37]	Chlorogenic acid 30, 60 mg/kg	Sprague-Dawley rats treated with CCl4 for eight weeks and CA for eight weeks	↓histological fibrosis ↓ collagen I, collagen III, α-SMA ↓ TGF-β, VEGF
Gressner OA, 2009 [26]	Caffeine metabolite (paraxanthine) 1.25 mM–2.5 mM	HSC treated with TGF-β + caffeine	↓ CTGF
Gressner OA, 2008 [25]	Caffeine 5 mM	Rat hepatocytes treated with cafeine	↓ CTGF, SMAD2 ↓ SMAD1/3-phosphorylation ↑ PPAR-γ
Chan ES, 2006 [24]	Caffeine 50 mg/kg daily orally	C57BL/six mice treated with CCl4 for six weeks or thioacetamide for seven weeks	↓ AST, ALT ↓ Picrosirius red staining

**Table 3:** Effects of coffee or coffee components in experimental models of liver cancer.

Author, Year	Coffee Compound	Study Design	Main Findings
Furtado KS, 2014 [39]	Regular Coffee Instant coffee Caffeine 0.1%	Wistar rats treated with DEN and CCl4 and administered coffee or caffeine for 24 weeks	↓ collagen I ↓ size and area of pre-neoplastic lesions ↓ number of neoplastic lesions
Ferk F, 2014 [40]	Coffee 25%, 50%, 100% coffee in drinking water	Rats administered coffee treated with aflatoxin B1 0.75 mg/kg b.w. ip and followed-up for 10 weeks	↓ number of pre-neoplastic foci for all brews ↑ UGT1A
Katayama M, 2014 [41]	Coffee	Long Evans Cinnamon rat administered coffee for 25 weeks	↑ survival ↓ liver inflammatory cytokines ↓ number of pre-neoplastic foci
Kalthoff S, 2010 [42]	Coffee	HepG2 and CaCo2 Transgenic mice expressing human UGT1A	↑ UGT1A isoforms in the liver
Higgins LC, 2008 [43]	Coffee 3% or 6%	Nrf2 (+/+) or Nrf2 (−/−) mice fed coffee for five days	↑ NQO1 and GSTA1 in the liver and gut of Nrf2 (+/+) mice ↑ UGT1A6 and GCLC in the gut of Nrf2 (+/+) mice
Cavin C, 2008 [44]	Coffee	Sprague-Dawley rats 0.75%; 1.5%; 3% or 6% coffee	↑ GSH, HO-1
Miura Y, 2004 [45]	Coffee	Hepatoma-bearing rats given oral administration of instant coffee powder (ICP) solution for two weeks	↓ tumor growth and metastases dissemination
Miura Y, 1997 [38]	Coffee	In vitro effects on a rat hepatoma cell line of sera from rats given oral administration of instant coffee powder (ICP) solution	↓ proliferation and invasion of hepatoma cells
Tanaka T, 1990 [34]	Coffee	Rats administered aminopyrine (0.01%) and sodium nitrite (0.1%) and contemporary drinking coffee solution for 630 days	↓ incidence of liver tumors ↓ number of hyperplastic liver cell foci
Mori H, 1986 [33]	Chlorogenic acid	Syrian golden hamsters given a single intravenous injection of MAM acetate (20 mg/kg body weight) and fed a diet containing 0.025% chlorogenic acid for 24 weeks	↓ incidence of colon tumors ↓ number of hyperplastic liver cell foci
